# Association between the -455T>C promoter polymorphism of the *APOC3 *gene and the metabolic syndrome in a multi-ethnic sample

**DOI:** 10.1186/1471-2350-8-80

**Published:** 2007-12-20

**Authors:** Rebecca L Pollex, Matthew R Ban, T Kue Young, Peter Bjerregaard, Sonia S Anand, Salim Yusuf, Bernard Zinman, Stewart B Harris, Anthony JG Hanley, Philip W Connelly, Murray W Huff, Robert A Hegele

**Affiliations:** 1 Vascular Biology Research Group, Robarts Research Institute, London, Ontario, Canada; 2 Department of Public Health Sciences, University of Toronto, Ontario, Canada; 3 National Institute of Public Health, Copenhagen, Denmark; 4 Population Health Research Institute, McMaster University, Hamilton Health Sciences, Hamilton, Ontario, Canada; 5 Department of Medicine, University of Toronto, and Samuel Lunenfeld Research Institute, Mount Sinai Hospital, Toronto, Ontario, Canada; 6 Thames Valley Family Practice Research Unit, University of Western Ontario, London, Ontario, Canada; 7 Department of Nutritional Sciences, University of Toronto, Toronto, Ontario, Canada; 8 Department of Laboratory Medicine and Pathobiology, University of Toronto, and the Keenan Research Centre, Li Ka Shing Knowledge Institute, St. Michael's Hospital, Toronto, Ontario, Canada; 9 Schulich School of Medicine and Dentistry, University of Western Ontario, London, Ontario, Canada

## Abstract

**Background:**

Common polymorphisms in the promoter of the *APOC3 *gene have been associated with hypertriglyceridemia and may impact on phenotypic expression of the metabolic syndrome (MetS). The rs7566605 marker, located near the *INSIG2 *gene, has been found to be associated with obesity, making it also a potential genetic determinant for MetS. The objective of this study is to examine the *APOC3 *-455T>C and the *INSIG2 *rs7566605 polymorphisms as potential genetic determinants for MetS in a multi-ethnic sample.

**Methods:**

Subjects were genotyped for both the *APOC3 *-455T>C and *INSIG2 *rs7566605 polymorphisms, and classified for the presence or absence of MetS (NCEP ATP III and IDF definitions). The total study population included 2675 subjects (≥18 years of age) from six different geographical ancestries.

**Results:**

For the overall study population, the prevalence of MetS was 22.6% (NCEP ATP III definition). Carriers of ≥1 copy of *APOC3 *-455C were more likely to have MetS (NCEP ATP III definition) than noncarriers (carrier odds ratio 1.73, 95% CI 1.40 to 2.14, adjusting for age and study group). The basis of the association was related not only to a higher proportion of -455C carriers meeting the triglyceride and high-density lipoprotein cholesterol criteria, but also the blood pressure criteria compared with wild-type homozygotes. Plasma apo C-III concentrations were not associated with *APOC3 *-455T>C genotype. The *INSIG2 *rs7566605  polymorphism was not associated with MetS or measures of obesity.

**Conclusion:**

Meta-analysis of the sample of multiple geographic ancestries indicated that the functional -455T>C promoter polymorphism in *APOC3 *was associated with an approximately 2-fold increased risk of MetS, whereas the *INSIG2 *rs7566605 polymorphism was not associated with MetS.

## Background

The metabolic syndrome (MetS) is a clinical entity characterized by abdominal obesity, hypertension, hypertriglyceridemia, low plasma high-density lipoprotein (HDL) cholesterol and elevated glucose [1,2]. MetS is common, and will likely become even more pervasive, considering the poor lifestyle habits prevalent in many societies today. While the increased prevalence in MetS is primarily related to an imbalance between caloric intake and expenditure, genetic factors are also likely to be important. Each defining component has been previously associated with genetic factors, suggesting that genetic factors might underlie the overall MetS both independently and through more complex interactions [3]. While the precise definition of MetS is controversial, there is no question that the MetS concept has proven to be valuable clinically [4,5].

A potential candidate underlying genetic susceptibility to MetS is apolipoprotein (apo) C-III (*APOC3*), one of the most studied genes in lipoprotein metabolism. *APOC3 *encodes a 79-amino-acid glycoprotein produced mainly in the liver, which acts as a constituent of triglyceride-rich lipoprotein particles, inhibiting the action of lipoprotein lipase and interfering with receptor-mediated lipoprotein uptake. The common -455T>C single nucleotide polymorphism (SNP) of the *APOC3 *gene, located within an insulin-response element in the promoter region, has previously been found to be associated with elevated plasma triglyceride concentrations in multiple populations [6-8].

Another potential candidate for MetS is the rs7566605 genetic variant, located 10 kb upstream of the insulin-induced gene 2 (*INSIG2*) gene, which was shown in a multi-staged genome-wide association study to be associated with obesity [9]. *INSIG2 *encodes a 225-aminoacid protein which blocks the proteolytic activation of sterol regulatory element-binding proteins [10], thus having antilipogenic effects, making it an attractive candidate for obesity-related traits. However, since the original report of an association between rs7566605 and BMI, results have been conflicting, with no association observed in four follow-up studies involving >22,000 subjects [11-14].

Thus, the purpose of this present study is to examine these two genetic polymorphisms: 1) the *APOC3 *-455T>C promoter polymorphism and 2) the rs7566605 SNP, located upstream of *INSIG2*, as potential genetic candidates for MetS, defined by both the National Cholesterol Education Program Adult Treatment Panel III (NCEP ATP III) and the International Diabetes Federation (IDF) criteria, in a sample of multiple geographic ancestries.

## Methods

### Study subjects

The multi-ethnic study included Inuit from Greenland and Canada (Kivalliq region, Nunavut), Oji-Cree (Ontario, Canada), and Canadians of South Asian, Chinese and European descent. All subjects for the current study were collected from one of the following population studies: 1) the Greenland Population Study [15]; 2) the Keewatin Health Assessment Study [16]; 3) the Sandy Lake Health and Diabetes Project [17]; and 4) the Study of Health Assessment and Risk in Ethnic Groups [18]. The details of these studies have been described previously [15-18]. Signed informed consent was obtained from all participants and the studies were approved by the relevant Ethics Review Committee. For the current analysis, the number of subjects ≥18 years of age with both sufficient DNA for *APOC3 *genotype determination and a complete set of data for MetS diagnosis included 1108 Greenland Inuit, 200 Canadian Inuit, 509 Oji-Cree, 327 South Asians, 299 Chinese, and 232 Caucasians; a total of 2675 subjects. There was no exclusion based on diabetes status. Fewer subjects had DNA available for *INSIG2 *genotyping.

### Physical measurements

Measurements of waist circumference, blood pressure, fasting analytes, including venous plasma glucose, serum cholesterol, triglycerides, low-density lipoprotein (LDL) cholesterol, and HDL cholesterol were performed as described [15-18]. For a subset of Greenland Inuit, plasma apo C-III concentrations were determined by a turbidimetric immunoassay (Wako Chemicals Inc., Richmond, VA).

### Metabolic syndrome classification

According to the National Cholesterol Education Program Adult Treatment Panel III (NCEP ATP III) criteria [1], MetS was identified if a subject had ≥3 of: 1) increased waist circumference (>102 cm [>40 inches] for men, >88 cm [>35 inches] for women); 2) elevated plasma triglycerides (≥1.69 mmol/L [≥150 mg/dL]); 3) low plasma HDL cholesterol (<1.04 mmol/L [<40 mg/dL] for men, <1.29 mmol/L [<50 mg/dL] for women); 4) hypertension (≥130/≥85 mmHg) or current medication; or 5) impaired fasting glucose (≥6.1 mmol/L [≥110 mg/dL]).

The International Diabetes Federation (IDF) criteria [19] identifies MetS for subjects with central obesity, according to ethnic specific guidelines (waist circumference for Europids ≥94 cm for men, ≥80 cm for women; for South Asians and Chinese ≥90 cm for men, ≥80 cm for women), plus any two of: 1) elevated plasma triglycerides (≥1.69 mmol/L [≥150 mg/dL]); 2) low plasma HDL cholesterol (<1.04 mmol/L [<40 mg/dL] for men, <1.29 mmol/L [<50 mg/dL] for women); 3) hypertension (≥130/≥85 mmHg) or current medication; or 4) impaired fasting glucose (≥5.6 mmol/L [≥100 mg/dL]) or previously diagnosed type 2 diabetes. Since no quantitative thresholds exist yet for aboriginal populations, these subjects were evaluated using the Chinese values for waist circumference.

### Genotyping of the *APOC3* and *INSIG2* polymorphisms

Detection of the *APOC3 *-455T>C promoter polymorphism (rs2854116) was carried out using a reported method [7]. Briefly, the primer set 5'-GTGAGAGCTCAGCCCCTGTAA-3' and 5'-TTTCACACTGGAAATTTCAGG-3' was used in a gene amplification program with annealing temperature 60°C to amplify a 194-bp fragment of the *APOC3 *promoter containing the insulin-response element. After *Fok*I (New England Biolabs, Mississauga, ON) digestion, the T allele yielded two fragments with sizes 122 and 72-bp, and the C allele yielded only a single 194-bp fragment. Electrophoresis in a 2.5% agarose gel followed by ethidium bromide staining and ultraviolet illumination allowed detection of the alleles.

Detection of the neighbouring *APOC3 *-482C>T promoter polymorphism (rs2854117) was carried out using either a reported method [7] or by using a custom designed TaqMan genotyping assay (Assay ID C_12081482_10; Applied Biosystems, Foster City, CA). The pairwise linkage disequilibrium correlation coefficient between the -455T>C and -482C>T SNPs across all samples was 0.89 (*P *< 0.0001), indicating strong linkage disequilibrium. Thus, for simplicity, considering the strong linkage disequilibrium observed, and the known functional impact of the DNA change, all subsequent analyses in the study were run using only the -455T>C SNP.

Detection of the rs7566605 SNP, located 10 kb upstream of the *INSIG2 *gene, was carried out using a validated TaqMan genotyping assay (Assay ID C_29404113_20; Applied Biosystems, Foster City, CA). SNP genotyping was performed using an allelic discrimination assay (TaqMan^® ^SNP Genotyping Assays, Applied Biosystems, Foster City, CA) using the 7900HT Fast Real-Time PCR System and genotypes were read using automated software (SDS 2.3, Applied Biosystems, Foster City, CA). Reactions were run in 5 μL volumes using an amplification protocol of 95°C for 10 minutes, followed by 50 cycles of 95°C for 15 seconds, then 60°C for 1.5 minutes. For all experiments, the genotyping success rate was in excess of 98%. When replicate quality control samples were evaluated, genotypes showed 100% concordance.

### Statistical analysis

SAS version 9.1 (SAS Institute, Cary, NC) was used for all statistical comparisons with analyses run separately for men and women. Data are presented as means ± standard deviation (s.d.) or as percentages for categorical variables. Differences in demographic and laboratory characteristics between groups were analyzed using either general linear or logistic models, adjusting for age. The *P*-values for blood pressure, cholesterol, triglycerides, LDL cholesterol, HDL cholesterol and glucose were also adjusted for BMI, in addition to age. Logistic regression analysis was also used to calculate the odds ratio (OR) for the MetS according to *APOC3 *-455T>C and rs7566605 genotype, adjusting for age and study group. The significance of deviations of observed genotype frequencies from those predicted by the Hardy-Weinberg equation were evaluated with χ^2 ^tests. The *APOC3 *-455T>C and rs7566605 G>C genotypes were included in the analysis as a dichotomous variable, in both dominant and recessive models. Statistical significance was taken at nominal *P*-value < 0.05 for all comparisons.

## Results

General characteristics of the six population groups included in the study are presented in Table S1 (see Additional file 1). Significant differences between the males and females are shown. Overall MetS prevalence, as defined by the NCEP ATP III criteria, was 14.9%, 13.5%, 35.4%, 32.4%, 19.4% and 29.3%, for the Greenland Inuit, Kivalliq Inuit, Oji-Cree, South Asians, Chinese and Caucasians, respectively. More females had MetS than males for the Greenland Inuit, Kivalliq Inuit and Oji-Cree groups (*P *= 0.017, 0.023, 0.028, respectively), no difference in MetS prevalence was observed between sex for the South Asian group, and more males had MetS than females for the Chinese and Caucasian groups (*P *= 0.0003 and 0.0078, respectively). The *APOC3 *-455C allele frequency was 0.41, 0.44, 0.46, 0.54, 0.44 and 0.41, for the Greenland Inuit, Kivalliq Inuit, Oji-Cree, South Asians, Chinese, and Caucasians, respectively. The *INSIG2 *rs7566605 C allele frequency was 0.20, 0.25, 0.22, 0.26, 0.36 and 0.31, for the Greenland Inuit, Kivalliq Inuit, Oji-Cree, South Asians, Chinese and Caucasians, respectively. The genotype frequencies did not deviate from the Hardy-Weinberg predictions (data not shown).

Table S2 (see Additional file 1) shows the demographic and metabolic characteristics of males and females based on *APOC3 *-455T>C genotype, assuming a dominant model for the C allele. Except for the Caucasian males, a trend towards elevated triglyceride concentrations was noted for -455C allele carriers in all groups, with significant increases for -455C allele carriers observed in both the Greenland Inuit females (*P *< 0.0001) and Oji-Cree females (*P *= 0.014), and close to significance for the Kivalliq Inuit females (*P *= 0.070). Significantly depressed HDL cholesterol concentrations were also observed for -455C allele carriers among Greenland Inuit females (*P *< 0.0001), Kivalliq Inuit females (*P *= 0.027), Oji-Cree females (*P *= 0.040) and Chinese males (*P *= 0.013). In addition, for Greenland Inuit females only, an association was found with MetS, with a greater prevalence of MetS observed for -455C allele carriers (20.3% *vs *11.1%, carrier OR 2.39, 95% confidence interval [CI] 1.44 to 3.98, *P *= 0.0008).

**Figure 1:**
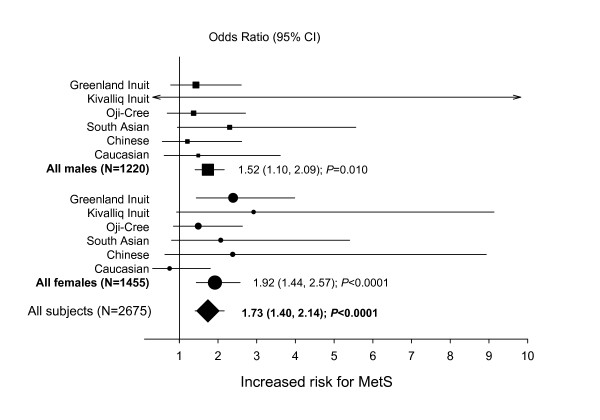
The age-adjusted odds ratios (ORs) and 95% confidence intervals (CI) for MetS in carriers of ≥1 copy of *APOC3 *-455C.

Meta-analysis of the 6 multi-ethnic study populations indicated that *APOC3 *-455C allele carriers had an increased risk of MetS (carrier OR 1.73, 95% CI 1.40 to 2.14, *P *< 0.0001) (Figure 1). Genotype and allele frequencies for the six study populations are shown in Table S3 (see Additional file 1). For females alone, the carrier OR was 1.92 (95% CI 1.44 to 2.57, *P *< 0.0001) and for males alone, the carrier OR was 1.52 (95% CI 1.10 to 2.09, *P *= 0.010). Repeating the analysis using a recessive model for the -455C allele indicated that CC homozygotes had an increased risk of MetS (OR 1.44, 95% CI 1.16 to 1.80, *P *= 0.0011). For females alone, the OR for CC homozygotes was 1.39 (95% CI 1.03 to 1.88, *P *= 0.031) and for males alone, the OR for CC homozygotes was 1.52 (95% CI 1.09 to 2.11, *P *= 0.012).

Meta-analysis of the overall sample indicated that significantly more *APOC3 *-455C allele carriers met the MetS criteria for elevated blood pressure (32.9% *vs *29.3%, *P *= 0.039), depressed HDL cholesterol (39.8% *vs *30.9%, *P *= 0.0002), and elevated triglycerides (*28.1% vs 18.6%*, *P *< 0.0001) than non-carriers, and tended to meet the criteria for fasting glucose (22.0% *vs *20.0%, *P *= NS[0.061]), but not for waist circumference (33.7% *vs *33.4%, *P *= NS[0.45]). This indicates that the association of the *APOC3 *-455C allele with the MetS was related in part to the lipid components, but also to non-lipid components, suggesting that this genetic marker may have a broader relationship with this complex trait.

Table S4 (see Additional file 1) shows the demographic and metabolic characteristics of males and females based on the *INSIG2 *rs7566605 G>C genotype, assuming a dominant model for the C allele. Significant differences between C allele carriers and non-carriers were observed for waist circumference measurements, with C allele carriers having a significantly smaller waist circumference for Oji-Cree females (*P *= 0.034), South Asian females (*P *= 0.013), and Chinese males (*P *= 0.012). Decreased waist circumference for C allele carriers among Caucasian females was close to significance (*P *= 0.067). No significant differences in the prevalence of MetS were observed for any of the populations depending on rs7566605 genotype.

**Figure 2:**
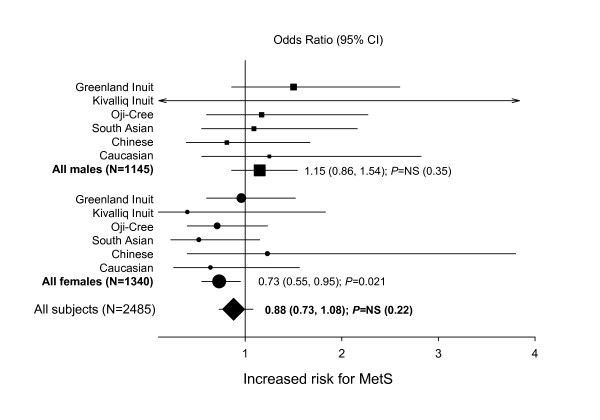
The age-adjusted odds ratios (ORs) and 95% confidence intervals (CI) for MetS in carriers of ≥1 copy of *INSIG2 *rs7566605 C.

Overall meta-analysis of the 6 multi-ethnic study populations indicated no significant association for the *INSIG2 *rs7566605 SNP with MetS (carrier OR 0.88, 95% CI 0.73 to 1.08, *P *= NS[0.22]) (Figure 2). Genotype and allele frequencies for the six study populations are shown in Table S5 (see Additional file 1). For females alone, the carrier OR was 0.73 (95% CI 0.55 to 0.95, *P *= 0.021) and for males alone, the carrier OR was 1.15 (95% CI 0.86 to 1.54, *P *= NS[0.35]). Repeating the analysis using a recessive model for the rs7566605 C allele indicated no significant association with MetS (OR 0.93, 95% CI 0.62 to 1.37, *P *= NS[0.70]). For females alone, the OR for CC homozygotes was 0.99 (95% CI 0.56 to 1.74, *P *= NS[0.97]) and for males alone, the OR for CC homozygotes was 0.87 (95% CI 0.50 to 1.50, *P *= NS[0.60]).

In the overall sample, carriers of the *INSIG2 *rs7566605 C "deleterious" allele were significantly *less likely *to meet the MetS criteria for elevated fasting glucose (*P *= 0.018) and increased waist circumference (*P *< 0.0001) than non-carriers. These findings directly contradict the initial findings that *INSIG2 *rs7566605 C is deleterious for obesity and related traits.

For comparison, associations between MetS factors and genotype were repeated, using the IDF definition of MetS. Once again, *APOC3 *-455C allele carriers had an increased risk of MetS (carrier OR 1.47, 95% CI 1.23 to 1.77, *P *< 0.0001). For females alone, the carrier OR was 1.85 (95% CI 1.43 to 2.39, *P *< 0.0001) and for males alone, the carrier OR was 1.14 (95% CI 0.87 to 1.48, *P *= NS[0.35]). Using a recessive model for the -455C allele indicated that CC homozygotes had an increased risk of MetS (OR 1.28, 95% CI 1.05 to 1.57, *P *= 0.017). For females alone, the OR for CC homozygotes was 1.30 (95% CI 0.98 to 1.72, *P *= NS[0.065]) and for males alone, the OR for CC homozygotes was 1.27 (95% CI 0.95 to 1.71, *P *= NS[0.11]).

Upon repeating the meta-analysis for the *INSIG2 *rs7566605 SNP with the IDF definition of MetS, a modest significant association – decreased risk – was found (carrier OR 0.83, 95% CI 0.69 to 0.99, *P *= 0.035). For females alone, the carrier OR was 0.64 (95% CI 0.50 to 0.82, *P *= 0.0004) and for males alone, the carrier OR was 1.11 (95% CI 0.86 to 1.43, *P *= NS[0.43]). Using a recessive model for the rs7566605 C allele indicated no significant association with MetS (OR 0.75, 95% CI 0.52 to 1.07, *P *= NS[0.11]). For females alone, the OR for CC homozygotes was 0.64 (95% CI 0.37 to 1.13, *P *= NS[0.13]) and for males alone, the OR for CC homozygotes was 0.83 (95% CI 0.51 to 1.35, *P *= NS[0.45]).

Plasma apo C-III concentrations were measured in a subset of 630 Greenland Inuit and analyzed according to *APOC3 *-455T>C genotype (Table S6, see Additional file 1). No significant differences in apoC-III concentration were observed based on genotype. ApoC-III concentrations for *APOC3 *-455C carriers *vs *non-carriers were 3.63 ± 1.76 mg/dL *vs *3.60 ± 2.26 mg/dL, *P *= NS(0.93), for the males, and 4.15 ± 2.15 mg/dL *vs *3.96 ± 2.07 mg/dL, *P *= NS(0.41), for the females. Plasma apo C-III concentrations were modestly correlated with triglycerides in this sample (*r *= 0.25, *P *< 0.0001).

## Discussion

Our study of candidate genes for MetS in a sample from multiple geographical ancestries showed 1) significant association with the *APOC3 *gene, with *APOC3 *-455C allele carriers having an increased risk of MetS (carrier OR 1.73, 95% CI 1.40 to 2.14; *P *< 0.0001); 2) this association was related to higher proportion of subjects with elevated triglyceride and depressed HDL cholesterol but also with lower blood pressure; and 3) no systematic associations of MetS phenotypes with genotypes of the *INSIG2 *rs7566605 marker; a few associations with *INSIG2 *in subgroups were statistically significant, but these were opposite to previously reported associations with respect to this disease phenotype.

The common promoter polymorphisms (-455T>C and -482C>T) of the *APOC3 *gene have been well established as bona fide functional variants, described as an example of insulin resistance at the gene level [20]. Studies on the transcriptional activity of promoter constructs containing either the -455C or -482T alleles, which are located within an insulin-responsive element (-490 to -449), have found that these variants are unable to respond effectively to insulin-mediated down-regulation and instead remain constitutively active [20]. Furthermore, the -455C variant has been found to have a reduced affinity for specific DNA binding proteins [20]. Consequently, considering apo C-III's inhibitory role towards lipoprotein lipase and the cellular uptake of triglyceride-rich lipoprotein particles, overexpression of apoC-III may promote the development of hypertriglyceridemia, as has been observed in overexpression studies in transgenic mice [21], and in human *APOC3 *promoter variant association studies [6-8].

Similarly, triglyceride concentrations were observed to be ~20% higher for -455C carriers (*P *< 0.0001 for Greenland Inuit, *P *= 0.014 for Oji-Cree), particularly in females, as predicted by the known loss of *APOC3 *transcriptional regulation associated with this allele. A significant lowering of HDL cholesterol concentrations was also observed among the female -455C carriers, suggesting further downstream disturbances in lipid metabolism, such as enhanced cholesteryl ester transfer protein activity or changes in the expression of the major HDL apolipoprotein, apoA-I, whose gene lies within the *APOA5*/*A4*/*C3*/*A1 *gene cluster [22]. Over-expression of apo C-III for carriers of the -455C variant however was not reflected in elevated apo C-III plasma concentrations upon examining a Greenland Inuit subset of the data, indicating that perhaps its effect may be intracellular or outside plasma. Conversely, increased concentrations of apo C-III were observed to be a common feature of the MetS-phenotype from a study of 563 Italian subjects, despite no association at the genetic level, questioning the mechanistic role of the *APOC3 *-455T>C genotype and suggesting another underlying mechanism, such as reduced protein catabolic rate for apo C-III, independent of increased apo C-III expression at the level of transcription [23]. Alternatively, the -455T>C SNP may be in linkage disequilibrium with another unmeasured genetic variant that was the actual determinant of the phenotypic association through an alternative mechanism. Clearly, this is a complex issue which remains to be clarified. Nonetheless, our data are consistent with the concept that *APOC3 *genotype may have a direct or indirect mechanistic role in the development of dyslipidemia and progression of MetS, and are in line with the recent report of a significant association with MetS in a small multi-ethnic study group [24].

More importantly, an association between the -455T>C *APOC3 *promoter polymorphism and MetS was observed following meta-analysis of the six multi-ethnic groups, with female carriers of ≥1 copy of *APOC3 *-455C found to have a significant ~2-fold increased risk for MetS (carrier OR 1.92, 95% CI 1.44 to 2.57; *P *< 0.0001), adjusting for age and study group, and male carriers having a significant ~1.5-fold increased risk (carrier OR 1.52, 95% CI 1.10 to 2.09; *P *= 0.010), resulting in an overall increased risk of ~1.7-fold (carrier OR 1.73, 95% CI 1.40 to 2.14; *P *< 0.0001). Under the IDF definition of MetS, the same trends were observed. The associations observed for the *APOC3 *-455T>C polymorphism with triglycerides and HDL cholesterol in these populations suggest that the association with the overall syndrome was mediated at least in part through association with some intermediate quantitative traits that are used in the definition of the syndrome. Indeed, in examining the relationship between the prevalence of each particular component of MetS and *APOC3 *-455T>C genotype, significant associations were found for the individual HDL cholesterol and triglyceride components (*P *= 0.0002 and <0.0001, respectively). Interestingly, a significant association was also found for the blood pressure component (*P *= 0.039) and fasting blood glucose was close to significance (*P *= 0.061). These findings suggest that the association of *APOC3 *genotype with MetS is related to associations with the two plasma lipoproteins and with additional non-lipoprotein components. Caucasian females did not follow the trend towards increased risk of MetS for -455C allele carriers suggesting that there may be gender-specific genetic factors associated with MetS which underlie the observed gender differences in MetS definition and prevalence, as observed previously [25,26].

Our study also examined the rs7566605 genetic variant, located 10 kb upstream of the *INSIG2 *gene, which was recently shown in a multi-staged genome-wide association study to be associated with obesity [9]. No association was observed for the rs7566605 marker with BMI, making this the fifth study [11-14] that fails to replicate the original association [9]. If anything, significant findings were in the opposite direction compared to the original publication, with waist circumference measurements significantly lower for carriers of the rare rs7566605 C allele in three of the groups studied. Also, no significant association was found for this variant with MetS, in individual populations, or in the combined meta-analysis (carrier OR 0.88, 95% CI 0.73 to 1.08, *P *= NS[0.22]). Furthermore, upon repeating the meta-analysis with the IDF definition of MetS, a modest but significant *decreased *risk was found overall for *INSIG2 *rs7566605 C allele carriers (carrier OR 0.83, 95% CI 0.69 to 0.99, *P *= 0.035), contrary to the expected direction of risk. These negative results again are a cautionary reminder of the great risk of false positive results in genome-wide association studies and stress the importance of replication and the use of functional markers [27].

## Conclusion

In summary, we report an association between MetS and a common promoter variant in *APOC3*. This association might be due to a direct effect of the genetic variation or to linkage disequilibrium with other functional changes. No association, however, was found for the *INSIG2 *rs7566605 marker with measures of obesity or with MetS. Finding a consistency in association between MetS and *APOC3 *in a multi-ethnic study group, including populations which differ considerably in MetS prevalence, strengthens the probability that the -455T>C *APOC3 *promoter polymorphism plays a key role in MetS expression. The findings suggest that factors involved in metabolism of triglyceride-rich lipoproteins may be important in the development of MetS and could represent markers for diagnosis or perhaps sample stratification for interventions.

## Competing interests

The author(s) declare that they have no competing interests.

## Authors' contributions

RLP participated in the experimental design, data acquisition and analysis, interpretation of results, and manuscript writing. MRB participated in the analysis of the data. TKY, PB, SSA, SY, BZ, SBH, AJGH, PWC and MWH were involved in the provision of patient samples and/or clinical data. SSA and MWH participated in the manuscript writing. RAH participated in the experimental design, data analysis and interpretation of results and manuscript writing. All authors approved the final manuscript.

## Pre-publication history

The pre-publication history for this paper can be accessed here:



## Supplementary Material

Additional file 1 Supplementary Tables. Six supplementary tables including: Table S1. Clinical and biochemical data of subjects. Table S2. Clinical and biochemical data of subjects when classified in accordance to their genotype of the *APOC3 *-455T>C polymorphism. Table S3. Genotype and allele frequencies for the *APOC3 *-455T>C polymorphism in subjects with and without MetS. Table S4. Clinical and biochemical data of subjects when classified in accordance to their genotype of the *INSIG2 *rs7566605 G>C polymorphism. Table S5. Genotype and allele frequencies for the *INSIG2 *rs7566605 G>C polymorphism in subjects with and without MetS. Table S6. Greenland Inuit plasma apo C-III concentration, by *APOC3 *-455T>C genotype Click here for file
