# Supplementary material for: Association between the -455T>C promoter polymorphism of the APOC3 gene and the metabolic syndrome in a multi-ethnic sample
Source: BMC Med Genet. 2007 Dec 20;8:80. doi: 10.1186/1471-2350-8-80 (PMC2241585; doi:10.1186/1471-2350-8-80)
Supplement: Additional file 1 — Supplementary Tables. Six supplementary tables including: Table S1. Clinical and biochemical data of subjects. Table S2. Clinical and biochemical data of subjects when classified in accordance to their genotype of the APOC3 -455T>C polymorphism. Table S3. Genotype and allele frequencies for the APOC3 -455T>C polymorphism in subjects with and without MetS. Table S4. Clinical and biochemical data of subjects when classified in accordance to their genotype of the INSIG2 rs7566605 G>C polymorphism. Table S5. Genotype and allele frequencies for the INSIG2 rs7566605 G>C polymorphism in subjects with and without MetS. Table S6. Greenland Inuit plasma apo C-III concentration, by APOC3 -455T>C genotype [file 1471-2350-8-80-S1.doc]

**Supplementary Tables**

**Table S1. Clinical and biochemical data of subjects**

|  | **Male** | **Female** | ***P*-value** |
| --- | --- | --- | --- |
| **Greenland Inuit** | **n = 486** | **n = 622** |  |
| age (years) | 46.2±12.9 | 45.8±13.1 | NS (0.60) |
| BMI (kg/m^2^) | 26.2±4.6 | 26.7±5.4^2^ | NS (0.072) |
| waist (cm) | 91.1±12.2 | 88.1±13.5 | 0.0001 |
| systolic BP (mmHg) | 120±17 | 119±20 | NS (0.14) |
| diastolic BP (mmHg) | 74±11 | 72±11 | <0.0001 |
| total cholesterol (mmol/L) | 5.99±1.18 | 6.02±1.13 | NS (0.77) |
| triglycerides (mmol/L) | 1.15±0.66 | 1.12±0.61 | NS (0.090) |
| LDL cholesterol (mmol/L) | 3.90±1.09^1^ | 3.89±1.06^2^ | NS (0.67) |
| HDL cholesterol (mmol/L) | 1.56±0.49 | 1.62±0.42 | 0.0015 |
| fasting glucose (mmol/L) | 5.83±0.89 | 5.76±1.34 | NS (0.21) |
| MetS (%) | 12.1 | 17.0 | 0.017 |
|  |  |  |  |
| **Kivalliq Inuit** | **n = 82** | **n = 118** |  |
| age (years) | 34.3±15.5 | 38.0±14.3 | NS (0.084) |
| BMI (kg/m^2^) | 25.9±4.0 | 27.1±4.7 | NS (0.15) |
| waist (cm) | 88.8±12.1 | 87.8±12.1 | NS (0.16) |
| systolic BP (mmHg) | 125±16 | 116±15 | <0.0001 |
| diastolic BP (mmHg) | 79±10 | 74±10 | <0.0001 |
| total cholesterol (mmol/L) | 4.75±1.04 | 5.02±0.95 | NS (0.28) |
| triglycerides (mmol/L) | 0.93±0.47 | 1.10±0.59 | NS (0.080) |
| LDL cholesterol (mmol/L) | 2.97±0.90 | 3.03±0.87 | NS (0.73) |
| HDL cholesterol (mmol/L) | 1.36±0.36 | 1.49±0.43 | 0.023 |
| fasting glucose (mmol/L) | 5.04±0.55 | 5.09±0.69 | NS (0.81) |
| MetS (%) | 6.1 | 18.6 | 0.023 |
|  |  |  |  |
| **Oji-Cree** | **n = 218** | **n = 291** |  |
| age (years) | 35.9±14.5 | 35.7±14.7 | NS (0.85) |
| BMI (kg/m^2^) | 26.8±4.6 | 29.1±5.5 | <0.0001 |
| waist (cm) | 96.9±12.2 | 95.0±11.9 | NS (0.073) |
| systolic BP (mmHg) | 122±14 | 118±16 | <0.0001 |
| diastolic BP (mmHg) | 70±12 | 67±10 | <0.0001 |
| total cholesterol (mmol/L) | 4.83±0.98 | 4.59±0.82 | 0.0001 |
| triglycerides (mmol/L) | 1.63±0.85 | 1.51±0.71 | 0.0003 |
| LDL cholesterol (mmol/L) | 2.91±0.82^3^ | 2.62±0.65^4^ | <0.0001 |
| HDL cholesterol (mmol/L) | 1.19±0.30 | 1.28±0.28 | <0.0001 |
| fasting glucose (mmol/L) | 6.73±3.25 | 6.77±3.50 | NS (0.61) |
| MetS (%) | 30.3 | 39.2 | 0.028 |
|  |  |  |  |
| **South Asian** | **n = 180** | **n = 147** |  |
| age (years) | 49.9±8.9 | 49.0±9.6 | NS (0.37) |
| BMI (kg/m^2^) | 26.1±4.3^5^ | 26.5±3.8 | NS (0.37) |
| waist (cm) | 94.7±9.7 | 86.0±10.6 | <0.0001 |
| systolic BP (mmHg) | 121±16 | 117±19 | 0.034 |
| diastolic BP (mmHg) | 80±12 | 71±10 | <0.0001 |
| total cholesterol (mmol/L) | 5.26±0.98 | 5.15±0.94 | NS (0.41) |
| triglycerides (mmol/L) | 2.05±1.31 | 1.92±1.27 | NS (0.30) |
| LDL cholesterol (mmol/L) | 3.45±0.84^6^ | 3.18±0.79^7^ | 0.0069 |
| HDL cholesterol (mmol/L) | 0.95±0.26 | 1.13±0.32 | <0.0001 |
| fasting glucose (mmol/L) | 5.95±1.89 | 5.49±1.58 | 0.020 |
| MetS (%) | 31.1 | 34.0 | NS (0.38) |
|  |  |  |  |
| **Chinese** | **n = 148** | **n = 151** |  |
| age (years) | 48.8±9.3 | 46.6±8.4 | 0.030 |
| BMI (kg/m^2^) | 25.2±3.4 | 22.7±3.5 | <0.0001 |
| waist (cm) | 89.6±9.1 | 75.2±7.9 | <0.0001 |
| systolic BP (mmHg) | 122±17 | 112±20 | 0.022 |
| diastolic BP (mmHg) | 80±10 | 70±12 | <0.0001 |
| total cholesterol (mmol/L) | 5.29±1.00 | 4.85±0.84 | 0.0057 |
| triglycerides (mmol/L) | 2.14±1.61 | 1.38±1.03 | 0.0055 |
| LDL cholesterol (mmol/L) | 3.34±0.84^8^ | 2.90±0.71^9^ | 0.0006 |
| HDL cholesterol (mmol/L) | 1.03±0.29 | 1.33±0.36 | <0.0001 |
| fasting glucose (mmol/L) | 5.44±1.29 | 4.98±0.60 | 0.016 |
| MetS (%) | 28.4 | 10.6 | 0.0003 |
|  |  |  |  |
| **Caucasian** | **n = 106** | **n = 126** |  |
| age (years) | 51.6±11.3 | 50.0±10.6 | NS (0.28) |
| BMI (kg/m^2^) | 28.3±4.0 | 26.6±4.8 | 0.0067 |
| waist (cm) | 100.4±12.6 | 84.5±12.1 | <0.0001 |
| systolic BP (mmHg) | 123±15 | 115±15 | 0.0008 |
| diastolic BP (mmHg) | 77±10 | 70±10 | <0.0001 |
| total cholesterol (mmol/L) | 5.18±0.96 | 4.98±0.87 | NS (0.40) |
| triglycerides (mmol/L) | 1.78±1.06 | 1.40±0.84 | 0.032 |
| LDL cholesterol (mmol/L) | 3.30±0.77^10^ | 2.99±0.77^11^ | 0.022 |
| HDL cholesterol (mmol/L) | 1.05±0.31 | 1.35±0.37 | <0.0001 |
| fasting glucose (mmol/L) | 5.71±2.00 | 5.14±1.23 | NS (0.065) |
| MetS (%) | 38.7 | 21.4 | 0.0078 |

^1^n=483, ^2^n=620, ^3^n=216, ^4^n=289, ^5^n=179, ^6^n=169, ^7^n=141, ^8^n=139, ^9^n=148, ^10^n=104, ^11^n=124

Abbreviations: BMI, body mass index; BP, blood pressure; LDL, low-density lipoprotein; HDL, high-density lipoprotein; MetS, metabolic syndrome; NS, not significant.

Data are means ± s.d. *P*-values are adjusted for age; *P*-values for blood pressure, cholesterol, triglycerides, LDL cholesterol, HDL cholesterol, and glucose are also adjusted for BMI.

# Table S2. Clinical and biochemical data of subjects when classified in accordance to their genotype of the *APOC3* –455T>C polymorphism

| **Characteristic** | **Male** | | |  | **Female** | | |
| --- | --- | --- | --- | --- | --- | --- | --- |
| **Greenland Inuit** | **T/T**  **n = 175** | **T/C & C/C**  **n = 311** | ***P*-value** |  | **T/T**  **n = 217** | **T/C & C/C**  **n = 405** | ***P*-value** |
| age (years) | 45.6±13.4 | 46.6±12.6 | NS (0.42) |  | 47.0±13.2 | 45.2±13.0 | NS (0.11) |
| BMI (kg/m^2^) | 25.7±4.3 | 26.4±4.7 | NS (0.12) |  | 26.6±5.1 | 26.8±5.6^4^ | NS (0.39) |
| waist (cm) | 90.6±11.5 | 91.4±12.5 | NS (0.58) |  | 87.6±13.3 | 88.3±13.6 | NS (0.22) |
| systolic BP (mmHg) | 120±17 | 120±17 | NS (0.22) |  | 119±21 | 119±20 | NS (0.34) |
| diastolic BP (mmHg) | 74±12 | 74±11 | NS (0.75) |  | 72±10 | 72±11 | NS (0.45) |
| total cholesterol (mmol/L) | 6.00±1.29 | 5.98±1.11 | NS (0.84) |  | 6.09±1.03 | 5.98±1.18 | NS (0.60) |
| triglycerides (mmol/L) | 1.12±0.70 | 1.16±0.63 | NS (0.68) |  | 0.99±0.42 | 1.19±0.69 | <0.0001 |
| LDL cholesterol (mmol/L) | 3.91±1.21^1^ | 3.89±1.01^2^ | NS (0.88) |  | 3.91±0.98^3^ | 3.88±1.11 | NS (0.63) |
| HDL cholesterol (mmol/L) | 1.57±0.50 | 1.56±0.48 | NS (0.99) |  | 1.73±0.43 | 1.57±0.41 | <0.0001 |
| fasting glucose (mmol/L) | 5.77±0.93 | 5.86±0.87 | NS (0.59) |  | 5.70±0.90 | 5.80±1.52 | NS (0.29) |
| MetS (%) | 9.7 | 13.5 | NS (0.22) |  | 11.1 | 20.3 | 0.0008 |
|  |  |  |  |  |  |  |  |
|  |  |  |  |  |  |  |  |
| **Kivalliq Inuit** | **n = 16** | **n = 66** |  |  | **n = 45** | **n = 73** |  |
| age (years) | 39.8±21.7 | 32.9±13.4 | NS (0.24) |  | 41.2±15.3 | 36.0±13.4 | NS (0.056) |
| BMI (kg/m^2^) | 25.5±4.4 | 26.0±3.9 | NS (0.45) |  | 27.0±5.0 | 27.2±4.5 | NS (0.38) |
| waist (cm) | 87.8±15.2 | 89.1±11.3 | NS (0.28) |  | 87.8±12.4 | 87.9±11.9 | NS (0.32) |
| systolic BP (mmHg) | 128±19 | 125±15 | NS (0.59) |  | 118±18 | 115±14 | NS (0.62) |
| diastolic BP (mmHg) | 75±11 | 80±10 | NS (0.15) |  | 74±11 | 73±9 | NS (0.55) |
| total cholesterol (mmol/L) | 4.54±1.09 | 4.80±1.03 | NS (0.081) |  | 5.17±1.02 | 4.93±0.90 | NS (0.49) |
| triglycerides (mmol/L) | 0.89±0.49 | 0.94±0.46 | NS (0.91) |  | 0.97±0.40 | 1.18±0.67 | NS (0.070) |
| LDL cholesterol (mmol/L) | 2.82±0.90 | 3.00±0.91 | NS (0.23) |  | 3.11±0.95 | 2.98±0.83 | NS (0.84) |
| HDL cholesterol (mmol/L) | 1.31±0.31 | 1.37±0.37 | NS (0.071) |  | 1.62±0.51 | 1.41±0.35 | 0.027 |
| fasting glucose (mmol/L) | 5.21±0.58 | 5.00±0.54 | NS (0.32) |  | 5.05±0.65 | 5.12±0.72 | NS (0.20) |
| MetS (%) | 0 | 7.6 | NS (0.96) |  | 13.3 | 21.9 | NS (0.066) |
|  |  |  |  |  |  |  |  |
|  |  |  |  |  |  |  |  |
|  |  |  |  |  |  |  |  |
| **Oji-Cree** | **n = 62** | **n = 156** |  |  | **n = 83** | **n = 208** |  |
| age (years) | 32.8±11.9 | 37.2±15.3 | 0.026 |  | 32.8±10.9 | 36.8±15.8 | 0.016 |
| BMI (kg/m^2^) | 26.2±4.4 | 27.0±4.7 | NS (0.58) |  | 29.3±6.0 | 29.0±5.3 | NS (0.48) |
| waist (cm) | 95.0±11.8 | 97.7±12.3 | NS (0.45) |  | 95.1±12.5 | 95.0±11.6 | NS (0.54) |
| systolic BP (mmHg) | 120±11 | 123±16 | NS (0.58) |  | 117±14 | 118±16 | NS (0.95) |
| diastolic BP (mmHg) | 68±10 | 71±13 | NS (0.43) |  | 67±10 | 67±10 | NS (0.59) |
| total cholesterol (mmol/L) | 4.65±0.93 | 4.91±1.00 | NS (0.30) |  | 4.43±0.71 | 4.66±0.85 | NS (0.12) |
| triglycerides (mmol/L) | 1.58±0.92 | 1.66±0.83 | NS (0.95) |  | 1.34±0.58 | 1.57±1.75 | 0.014 |
| LDL cholesterol (mmol/L) | 2.74±0.80^5^ | 2.98±0.83^6^ | NS (0.20) |  | 2.50±0.57 | 2.67±0.67 | NS (0.14) |
| HDL cholesterol (mmol/L) | 1.19±0.30 | 1.19±0.30 | NS (0.74) |  | 1.32±0.33 | 1.27±0.26 | 0.040 |
| fasting glucose (mmol/L) | 6.13±2.34 | 6.97±3.52 | NS (0.25) |  | 6.66±3.80 | 6.81±3.38 | NS (0.98) |
| MetS (%) | 24.2 | 32.7 | NS (0.37) |  | 30.1 | 42.8 | NS (0.16) |
|  |  |  |  |  |  |  |  |
|  |  |  |  |  |  |  |  |
| **South Asian** | **n = 41** | **n = 139** |  |  | **n = 32** | **n = 115** |  |
| age (years) | 49.7±8.9 | 49.9±8.9 | NS (0.89) |  | 48.2±7.8 | 49.2±10.1 | NS (0.62) |
| BMI (kg/m^2^) | 25.6±3.9 | 26.3±4.4^8^ | NS (0.34) |  | 25.8±3.9 | 26.7±3.8 | NS (0.21) |
| waist (cm) | 94.1±9.1 | 94.8±9.9 | NS (0.67) |  | 85.3±10.7 | 86.3±10.6 | NS (0.74) |
| systolic BP (mmHg) | 121±17 | 121±15 | NS (0.90) |  | 115±18 | 118±20 | NS (0.78) |
| diastolic BP (mmHg) | 79±10 | 80±12 | NS (0.80) |  | 69±9 | 72±10 | NS (0.48) |
| total cholesterol (mmol/L) | 5.13±0.98 | 5.29±0.98 | NS (0.40) |  | 5.25±0.99 | 5.12±0.93 | NS (0.32) |
| triglycerides (mmol/L) | 1.77±1.01 | 2.13±1.38 | NS (0.15) |  | 1.62±0.87 | 2.00±1.35 | NS (0.24) |
| LDL cholesterol (mmol/L) | 3.34±0.93^7^ | 3.48±0.81^9^ | NS (0.44) |  | 3.38±0.90^10^ | 3.12±0.75^11^ | NS (0.083) |
| HDL cholesterol (mmol/L) | 0.99±0.31 | 0.93±0.244 | NS (0.29) |  | 1.13±0.24 | 1.13±0.34 | NS (0.99) |
| fasting glucose (mmol/L) | 6.17±1.99 | 5.89±1.86 | NS (0.35) |  | 5.27±1.04 | 5.55±1.70 | NS (0.60) |
| MetS (%) | 18.9 | 34.3 | NS (0.064) |  | 21.9 | 37.4 | NS (0.14) |
|  |  |  |  |  |  |  |  |
|  |  |  |  |  |  |  |  |
|  |  |  |  |  |  |  |  |
|  |  |  |  |  |  |  |  |
|  |  |  |  |  |  |  |  |
|  |  |  |  |  |  |  |  |
|  |  |  |  |  |  |  |  |
|  |  |  |  |  |  |  |  |
| **Chinese** | **n = 50** | **n = 98** |  |  | **n = 48** | **n = 103** |  |
| age (years) | 49.3±9.6 | 48.6±9.1 | NS (0.66) |  | 47.6±9.3 | 46.1±8.0 | NS (0.30) |
| BMI (kg/m^2^) | 25.3±4.0 | 25.1±3.1 | NS (0.75) |  | 22.2±3.0 | 23.0±3.6 | NS (0.17) |
| waist (cm) | 89.9±10.5 | 89.4±8.3 | NS (0.73) |  | 74.4±7.8 | 75.6±8.0 | NS (0.32) |
| systolic BP (mmHg) | 121±16 | 123±17 | NS (0.39) |  | 111±20 | 113±20 | NS (0.45) |
| diastolic BP (mmHg) | 77±10 | 81±10 | 0.0091 |  | 68±10 | 71±13 | NS (0.10) |
| total cholesterol (mmol/L) | 5.20±0.98 | 5.34±1.01 | NS (0.41) |  | 4.78±0.78 | 4.88±0.86 | NS (0.34) |
| triglycerides (mmol/L) | 2.01±2.06 | 2.20±1.33 | NS (0.43) |  | 1.20±1.06 | 1.47±1.01 | NS (0.19) |
| LDL cholesterol (mmol/L) | 3.25±0.85^12^ | 3.39±0.84^13^ | NS (0.34) |  | 2.95±0.71^14^ | 2.89±0.71^15^ | NS (0.70) |
| HDL cholesterol (mmol/L) | 1.10±0.32 | 0.99±0.27 | 0.013 |  | 1.33±0.28 | 1.33±0.39 | NS (0.62) |
| fasting glucose (mmol/L) | 5.47±1.61 | 5.43±1.10 | NS (0.91) |  | 4.86±0.47 | 5.03±0.64 | NS (0.19) |
| MetS (%) | 26.0 | 29.6 | NS (0.63) |  | 6.25 | 12.6 | NS (0.20) |
|  |  |  |  |  |  |  |  |
| **Caucasian** | **n = 35** | **n = 71** |  |  | **n = 47** | **n = 79** |  |
| age (years) | 50.3±10.9 | 52.2±11.5 | NS (0.41) |  | 51.4±11.4 | 49.2±10.1 | NS (0.27) |
| BMI (kg/m^2^) | 28.5±4.0 | 28.2±4.0 | NS (0.65) |  | 27.4±5.5 | 26.1±4.4 | NS (0.19) |
| waist (cm) | 101±12 | 100±13 | NS (0.71) |  | 87.1±13.5 | 82.9±11.0 | NS (0.077) |
| systolic BP (mmHg) | 123±17 | 124±15 | NS (0.89) |  | 119±16 | 112±14 | NS (0.11) |
| diastolic BP (mmHg) | 75±10 | 78±10 | NS (0.092) |  | 73±10 | 69±9 | 0.032 |
| total cholesterol (mmol/L) | 5.26±1.05 | 5.13±0.91 | NS (0.58) |  | 5.01±0.83 | 4.96±0.91 | NS (0.69) |
| triglycerides (mmol/L) | 1.91±1.19 | 1.72±0.99 | NS (0.47) |  | 1.37±0.74 | 1.42±0.89 | NS (0.23) |
| LDL cholesterol (mmol/L) | 3.30±0.90^16^ | 3.33±0.77^17^ | NS (0.71) |  | 3.04±0.71^18^ | 2.96±0.81^19^ | NS (0.97) |
| HDL cholesterol (mmol/L) | 1.07±0.36 | 1.04±0.28 | NS (0.44) |  | 1.36±0.39 | 1.35±0.35 | NS (0.66) |
| fasting glucose (mmol/L) | 5.86±2.58 | 5.64±1.66 | NS (0.47) |  | 5.21±1.30 | 5.10±1.19 | NS (0.82) |
| MetS (%) | 31.4 | 42.3 | NS (0.27) |  | 25.5 | 19.0 | NS (0.52) |

^1^n=173, ^2^n=310, ^3^n= 403, ^4^n=215, ^5^n=61, ^6^n=155, ^7^n=40, ^8^n=138, ^9^n=129, ^10^n=30, ^11^n=110, ^12^n=47, ^13^n=92, ^14^n=47, ^15^n=101, ^16^n=34, ^17^n=70, ^18^n=46, ^19^n=78

Abbreviations: BMI, body mass index; BP, blood pressure; LDL, low-density lipoprotein; HDL, high-density lipoprotein; MetS, metabolic syndrome; NS, not significant.

Data are means ± s.d. *P*-values are adjusted for age; *P*-values for blood pressure, cholesterol, triglycerides, LDL cholesterol, HDL cholesterol, and glucose are also adjusted for BMI.

# Table S3. Genotype and allele frequencies for the *APOC3* -455T>C polymorphism in subjects with and without MetS

| **Population** | **Genotype frequencies** | | |  | **Allele frequencies** | |  | ***P*-value** | | |
| --- | --- | --- | --- | --- | --- | --- | --- | --- | --- | --- |
| **Greenland Inuit** | **T/T** | **T/C** | **C/C** |  | **T** | **C** |  | **Dominant model** | **Co-dominant model** | **Recessive model** |
| With MetS | 0.25 | 0.56 | 0.19 |  | 0.53 | 0.47 |  |  |  |  |
| Without MetS | 0.37 | 0.46 | 0.17 |  | 0.60 | 0.40 |  | 0.0011 | 0.0044 | NS (0.49) |
|  |  |  |  |  |  |  |  |  |  |  |
| **Kivalliq Inuit** |  |  |  |  |  |  |  |  |  |  |
| With MetS | 0.22 | 0.52 | 0.26 |  | 0.48 | 0.52 |  |  |  |  |
| Without MetS | 0.32 | 0.52 | 0.16 |  | 0.58 | 0.42 |  | NS (0.11) | NS (0.25) | NS (0.31) |
|  |  |  |  |  |  |  |  |  |  |  |
| **Oji-Cree** |  |  |  |  |  |  |  |  |  |  |
| With MetS | 0.22 | 0.51 | 0.27 |  | 0.48 | 0.52 |  |  |  |  |
| Without MetS | 0.32 | 0.52 | 0.16 |  | 0.58 | 0.42 |  | NS (0.11) | 0.023 | 0.0089 |
|  |  |  |  |  |  |  |  |  |  |  |
| **South Asian** |  |  |  |  |  |  |  |  |  |  |
| With MetS | 0.14 | 0.46 | 0.40 |  | 0.37 | 0.63 |  |  |  |  |
| Without MetS | 0.26 | 0.47 | 0.27 |  | 0.50 | 0.50 |  | 0.017 | 0.021 | 0.026 |
|  |  |  |  |  |  |  |  |  |  |  |
| **Chinese** |  |  |  |  |  |  |  |  |  |  |
| With MetS | 0.28 | 0.55 | 0.17 |  | 0.55 | 0.45 |  |  |  |  |
| Without MetS | 0.34 | 0.45 | 0.21 |  | 0.56 | 0.44 |  | NS (0.29) | NS (0.32) | NS (0.52) |
|  |  |  |  |  |  |  |  |  |  |  |
| **Caucasian** |  |  |  |  |  |  |  |  |  |  |
| With MetS | 0.34 | 0.49 | 0.18 |  | 0.58 | 0.42 |  |  |  |  |
| Without MetS | 0.36 | 0.48 | 0.16 |  | 0.60 | 0.40 |  | NS (0.71) | NS (0.58) | NS (0.30) |

*P*-values are adjusted for age.

# Table S4. Clinical and biochemical data of subjects when classified in accordance to their genotype of the *INSIG2* rs7566605 G>C polymorphism

| **Characteristic** | **Male** | | |  | **Female** | | |
| --- | --- | --- | --- | --- | --- | --- | --- |
| **Greenland Inuit** | **G/G**  **n = 300** | **G/C & C/C**  **n = 180** | ***P*-value** |  | **G/G**  **n = 401** | **G/C & C/C**  **n = 212** | ***P*-value** |
| age (years) | 46.4±13.0 | 46.2±12.7 | NS (0.88) |  | 45.9±13.1 | 45.7±13.1 | NS (0.88) |
| BMI (kg/m^2^) | 26.1±4.6 | 26.4±4.5 | NS (0.42) |  | 26.8±5.4^3^ | 26.5±5.3^4^ | NS (0.48) |
| waist (cm) | 91.0±12.4 | 91.5±11.9 | NS (0.64) |  | 88.4±13.3 | 87.5±13.7 | NS (0.46) |
| systolic BP (mmHg) | 120±17 | 120±18 | NS (0.95) |  | 119±21 | 118±19 | NS (0.34) |
| diastolic BP (mmHg) | 74±12 | 75±11 | NS (0.81) |  | 72±14 | 71±11 | NS (0.17) |
| total cholesterol (mmol/L) | 6.02±1.17 | 5.93±1.19 | NS (0.38) |  | 6.07±1.15 | 5.92±1.08 | NS (0.15) |
| triglycerides (mmol/L) | 1.13±0.67 | 1.17±0.64 | NS (0.70) |  | 1.12±0.62 | 1.09±0.58 | NS (0.76) |
| LDL cholesterol (mmol/L) | 3.93±1.08^1^ | 3.84±1.10^2^ | NS (0.30) |  | 3.94±1.08^3^ | 3.79±1.02^4^ | NS (0.11) |
| HDL cholesterol (mmol/L) | 1.57±0.47 | 1.56±0.51 | NS (0.86) |  | 1.61±0.44 | 1.64±0.39 | NS (0.64) |
| fasting glucose (mmol/L) | 5.81±0.88 | 5.84±0.90 | NS (0.84) |  | 5.79±1.49 | 5.71±1.00 | NS (0.58) |
| MetS (%) | 10.7 | 15.0 | NS (0.15) |  | 17.2 | 16.5 | NS (0.85) |
|  |  |  |  |  |  |  |  |
|  |  |  |  |  |  |  |  |
| **Kivalliq Inuit** | **n = 37** | **n = 22** |  |  | **n = 36** | **n = 33** |  |
| age (years) | 33.3±16.0 | 42.2±16.8 | 0.048 |  | 39.0±16.3 | 40.2±12.9 | NS (0.72) |
| BMI (kg/m^2^) | 26.4±3.8 | 26.1±4.3 | NS (0.44) |  | 27.0±4.7 | 27.6±5.1 | NS (0.66) |
| waist (cm) | 89.7±12.1 | 90.0±13.1 | NS (0.40) |  | 88.5±11.7 | 89.3±13.8 | NS (0.91) |
| systolic BP (mmHg) | 128±21 | 124±17 | NS (0.15) |  | 116±14 | 118±16 | NS (0.65) |
| diastolic BP (mmHg) | 81±9 | 77±10 | NS (0.39) |  | 73±8 | 73±10 | NS (0.89) |
| total cholesterol (mmol/L) | 4.75±1.17 | 5.11±0.98 | NS (0.55) |  | 5.11±1.09 | 5.30±1.06 | NS (0.55) |
| triglycerides (mmol/L) | 0.90±0.48 | 0.98±0.46 | NS (0.27) |  | 1.18±0.64 | 1.14±0.55 | NS (0.66) |
| LDL cholesterol (mmol/L) | 2.92±0.95 | 3.29±0.95 | NS (0.26) |  | 3.04±0.98 | 3.29±0.99 | NS (0.37) |
| HDL cholesterol (mmol/L) | 1.42±0.44 | 1.37±0.29 | NS (0.051) |  | 1.53±0.51 | 1.49±0.35 | NS (0.80) |
| fasting glucose (mmol/L) | 5.16±0.51 | 5.12±0.56 | NS (0.33) |  | 4.93±0.74 | 5.27±0.82 | NS (0.084) |
| MetS (%) | 8.1 | 0 | NS (0.96) |  | 19.4 | 9.1 | NS (0.24) |
|  |  |  |  |  |  |  |  |
|  |  |  |  |  |  |  |  |
|  |  |  |  |  |  |  |  |
| **Oji-Cree** | **n = 113** | **n = 67** |  |  | **n = 142** | **n = 104** |  |
| age (years) | 34.7±13.6 | 37.0±15.2 | NS (0.29) |  | 37.1±15.2 | 33.3±13.1 | 0.040 |
| BMI (kg/m^2^) | 26.6±4.8 | 26.5±4.6 | NS (0.57) |  | 29.5±5.4 | 28.3±5.4 | NS (0.18) |
| waist (cm) | 96.4±12.6 | 96.4±11.9 | NS (0.66) |  | 96.8±11.9 | 92.6±11.9 | 0.034 |
| systolic BP (mmHg) | 121±14 | 123±14 | NS (0.63) |  | 119±16 | 117±16 | NS (0.62) |
| diastolic BP (mmHg) | 69±12 | 72±12 | NS (0.15) |  | 67±9 | 66±12 | NS (0.57) |
| total cholesterol (mmol/L) | 4.79±0.92 | 4.89±1.02 | NS (0.61) |  | 4.56±0.75 | 4.60±0.88 | NS (0.14) |
| triglycerides (mmol/L) | 1.59±0.82 | 1.68±0.89 | NS (0.36) |  | 1.54±0.68 | 1.45±0.74 | NS (0.76) |
| LDL cholesterol (mmol/L) | 2.86±0.80^5^ | 2.93±0.87 | NS (0.70) |  | 2.56±0.62^6^ | 2.67±0.68^7^ | 0.021 |
| HDL cholesterol (mmol/L) | 1.21±0.29 | 1.20±0.32 | NS (0.56) |  | 1.29±0.28 | 1.25±0.27 | NS (0.24) |
| fasting glucose (mmol/L) | 6.80±3.17 | 6.81±3.48 | NS (0.80) |  | 6.79±3.18 | 6.67±3.82 | NS (0.68) |
| MetS (%) | 28.3 | 32.8 | NS (0.65) |  | 46.5 | 34.6 | NS (0.22) |
|  |  |  |  |  |  |  |  |
|  |  |  |  |  |  |  |  |
| **South Asian** | **n = 93** | **n = 79** |  |  | **n = 84** | **n = 58** |  |
| age (years) | 48.9±8.5 | 51.0±9.5 | NS (0.13) |  | 48.7±9.4 | 49.4±9.8 | NS (0.66) |
| BMI (kg/m^2^) | 26.4±4.7 | 26.0±3.8^9^ | NS (0.53) |  | 26.7±3.9 | 26.0±3.7 | NS (0.26) |
| waist (cm) | 95.0±8.9 | 94.9±10.2 | NS (0.65) |  | 87.6±10.2 | 83.4±11.1 | 0.013 |
| systolic BP (mmHg) | 119±15 | 124±16 | NS (0.12) |  | 117±18 | 118±20 | NS (0.71) |
| diastolic BP (mmHg) | 78±9 | 81±15 | NS (0.071) |  | 71±10 | 72±10 | NS (0.33) |
| total cholesterol (mmol/L) | 5.24±0.96 | 5.24±1.02 | NS (0.84) |  | 5.06±0.96 | 5.23±0.91 | NS (0.34) |
| triglycerides (mmol/L) | 2.10±1.52 | 1.95±1.02 | NS (0.53) |  | 1.98±1.46 | 1.81±0.96 | NS (0.53) |
| LDL cholesterol (mmol/L) | 3.46±0.85^8^ | 3.43±0.85^10^ | NS (0.71) |  | 3.12±0.84^11^ | 3.24±0.71^12^ | NS (0.44) |
| HDL cholesterol (mmol/L) | 0.94±0.27 | 0.96±0.25 | NS (0.76) |  | 1.10±0.30 | 1.13±0.34 | NS (0.35) |
| fasting glucose (mmol/L) | 6.04±2.05 | 5.88±1.75 | NS (0.34) |  | 5.52±1.41 | 5.46±1.85 | NS (0.94) |
| MetS (%) | 28.0 | 32.9 | NS (0.81) |  | 38.1 | 27.6 | NS (0.11) |
|  |  |  |  |  |  |  |  |
|  |  |  |  |  |  |  |  |
|  |  |  |  |  |  |  |  |
|  |  |  |  |  |  |  |  |
|  |  |  |  |  |  |  |  |
|  |  |  |  |  |  |  |  |
|  |  |  |  |  |  |  |  |
|  |  |  |  |  |  |  |  |
| **Chinese** | **n = 70** | **n = 77** |  |  | **n = 50** | **n = 96** |  |
| age (years) | 48.2±9.2 | 49.2±9.3 | NS (0.52) |  | 47.7±8.4 | 46.2±8.6 | NS (0.30) |
| BMI (kg/m^2^) | 25.6±3.7 | 24.8±3.1 | NS (0.21) |  | 22.7±4.0 | 22.8±3.2 | NS (0.86) |
| waist (cm) | 91.6±10.0 | 87.9±7.7 | 0.012 |  | 75.5±9.1 | 75.0±7.4 | NS (0.82) |
| systolic BP (mmHg) | 122±16 | 122±18 | NS (0.85) |  | 113±17 | 112±22 | NS (0.80) |
| diastolic BP (mmHg) | 80±10 | 80±11 | NS (0.44) |  | 70±11 | 70±12 | NS (0.76) |
| total cholesterol (mmol/L) | 5.24±1.00 | 5.36±1.01 | NS (0.45) |  | 5.02±0.87 | 4.81±0.78 | NS (0.24) |
| triglycerides (mmol/L) | 2.27±1.87 | 2.04±1.34 | NS (0.51) |  | 1.43±0.89 | 1.37±1.12 | NS (0.81) |
| LDL cholesterol (mmol/L) | 3.26±0.79^13^ | 3.43±0.89^14^ | NS (0.22) |  | 2.99±0.74^15^ | 2.90±0.68^16^ | NS (0.59) |
| HDL cholesterol (mmol/L) | 1.01±0.31 | 1.05±0.28 | NS (0.73) |  | 1.37±0.40 | 1.33±0.34 | NS (0.52) |
| fasting glucose (mmol/L) | 5.55±1.43 | 5.34±1.16 | NS (0.36) |  | 5.05±0.73 | 4.95±0.53 | NS (0.31) |
| MetS (%) | 30.0 | 26.0 | NS (0.56) |  | 10.0 | 11.5 | NS (0.72) |
|  |  |  |  |  |  |  |  |
| **Caucasian** | **n = 47** | **n = 60** |  |  | **n = 59** | **n = 65** |  |
| age (years) | 53.1±11.4 | 51.9±11.7 | NS (0.61) |  | 49.6±10.5 | 50.0±10.6 | NS (0.85) |
| BMI (kg/m^2^) | 28.3±4.4 | 27.9±3.4 | NS (0.56) |  | 27.0±5.2 | 26.4±5.0 | NS (0.47) |
| waist (cm) | 101±14 | 99±11 | NS (0.57) |  | 86.5±12.7 | 82.5±11.7 | NS (0.067) |
| systolic BP (mmHg) | 122±17 | 124±15 | NS (0.27) |  | 114±13 | 116±17 | NS (0.36) |
| diastolic BP (mmHg) | 77±9 | 78±10 | NS (0.54) |  | 70±10 | 71±10 | NS (0.17) |
| total cholesterol (mmol/L) | 5.18±0.88 | 5.16±1.02 | NS (0.99) |  | 4.95±0.88 | 4.89±0.80 | NS (0.73) |
| triglycerides (mmol/L) | 1.69±0.82 | 1.80±1.18 | NS (0.47) |  | 1.49±0.92 | 1.28±0.73 | NS (0.17) |
| LDL cholesterol (mmol/L) | 3.34±0.74 | 3.31±0.87^17^ | NS (0.99) |  | 2.92±0.80^17^ | 2.96±0.67 | NS (0.69) |
| HDL cholesterol (mmol/L) | 1.07±0.30 | 1.04±0.33 | NS (0.50) |  | 1.35±0.42 | 1.34±0.31 | NS (0.66) |
| fasting glucose (mmol/L) | 5.59±1.48 | 5.78±2.34 | NS (0.46) |  | 5.34±1.55 | 4.92±0.82 | NS (0.064) |
| MetS (%) | 36.2 | 40.0 | NS (0.68) |  | 23.7 | 16.9 | NS (0.32) |

^1^n=298, ^2^n=179, ^3^n=400, ^4^n=211, ^5^n=112, ^6^n=141, ^7^n=103, ^8^n=86, ^i^n=78, ^j^n=75, ^k^n=80, ^l^n=56, ^m^n=65, ^n^n=73, ^o^n=49, ^p^n=94, ^17^n=58

Abbreviations: BMI, body mass index; BP, blood pressure; LDL, low-density lipoprotein; HDL, high-density lipoprotein; MetS, metabolic syndrome; NS, not significant.

Data are means ± s.d. *P*-values are adjusted for age; *P*-values for blood pressure, cholesterol, triglycerides, LDL cholesterol, HDL cholesterol, and glucose are also adjusted for BMI.

# Table S5. Genotype and allele frequencies for the *INSIG2* rs7566605 G>C polymorphism in subjects with and without MetS

| **Population** | **Genotype frequencies** | | |  | **Allele frequencies** | |  | ***P*-value** | | |
| --- | --- | --- | --- | --- | --- | --- | --- | --- | --- | --- |
| **Greenland Inuit** | **G/G** | **G/C** | **C/C** |  | **G** | **C** |  | **Dominant model** | **Co-dominant model** | **Recessive model** |
| With MetS | 0.62 | 0.36 | 0.02 |  | 0.80 | 0.20 |  |  |  |  |
| Without MetS | 0.65 | 0.30 | 0.05 |  | 0.80 | 0.20 |  | NS (0.49) | NS (0.15) | NS (0.12) |
|  |  |  |  |  |  |  |  |  |  |  |
| **Kivalliq Inuit** |  |  |  |  |  |  |  |  |  |  |
| With MetS | 0.77 | 0.15 | 0.08 |  | 0.85 | 0.15 |  |  |  |  |
| Without MetS | 0.55 | 0.37 | 0.08 |  | 0.74 | 0.26 |  | NS (0.087) | NS (0.20) | NS (0.93) |
|  |  |  |  |  |  |  |  |  |  |  |
| **Oji-Cree** |  |  |  |  |  |  |  |  |  |  |
| With MetS | 0.63 | 0.33 | 0.04 |  | 0.80 | 0.20 |  |  |  |  |
| Without MetS | 0.58 | 0.37 | 0.05 |  | 0.77 | 0.23 |  | NS (0.47) | NS (0.70) | NS (0.54) |
|  |  |  |  |  |  |  |  |  |  |  |
| **South Asian** |  |  |  |  |  |  |  |  |  |  |
| With MetS | 0.58 | 0.32 | 0.10 |  | 0.74 | 0.26 |  |  |  |  |
| Without MetS | 0.56 | 0.38 | 0.07 |  | 0.75 | 0.25 |  | NS (0.33) | NS (0.43) | NS (0.62) |
|  |  |  |  |  |  |  |  |  |  |  |
| **Chinese** |  |  |  |  |  |  |  |  |  |  |
| With MetS | 0.46 | 0.37 | 0.18 |  | 0.64 | 0.36 |  |  |  |  |
| Without MetS | 0.40 | 0.48 | 0.12 |  | 0.64 | 0.36 |  | NS (0.45) | NS (0.35) | NS (0.37) |
|  |  |  |  |  |  |  |  |  |  |  |
| **Caucasian** |  |  |  |  |  |  |  |  |  |  |
| With MetS | 0.47 | 0.44 | 0.09 |  | 0.69 | 0.31 |  |  |  |  |
| Without MetS | 0.45 | 0.48 | 0.06 |  | 0.70 | 0.30 |  | NS (0.85) | NS (0.62) | NS (0.38) |

# *P*-values are adjusted for age.

# Table S6. Greenland Inuit plasma apo C-III concentration, by *APOC3* -455T>C genotype

| ***Sex*** | **APOC3 *genotype***  ***(-455T>C)*** | ***N*** | ***apo C-III (mg/dL)*** | **P*-value***  ***T/T* vs *T/C & C/C*** |
| --- | --- | --- | --- | --- |
| Male | T/T | 123 | 3.60±2.26 | NS (0.93) |
|  | T/C | 123 | 3.85±1.67 |  |
|  | C/C | 69 | 3.23±1.85 |  |
|  |  |  |  |  |
| Female | T/T | 123 | 3.96±2.07 | NS (0.41) |
|  | T/C | 123 | 4.14±2.01 |  |
|  | C/C | 69 | 4.16±2.39 |  |

Data are means ± s.d. *P*-values are adjusted for age, BMI.
